# Effervescing Powders

**Published:** 1857-01

**Authors:** Jno. M. Maisch

**Affiliations:** Philadelphia


### SELECTIONS.

From Proceedings of American Pharmaceutical Association.

### *On Effervescing Powders.* By Jno. M. Maisch, of Philadelphia.

Effervescing powders are used on account of the carbonic acid in gaseous state which is generated as soon as they are thrown into water to dissolve. The carbonic acid is highly esteemed for its agreeable refrigerance, or it is employed to mask to a certain extent the taste of other medicines. The generation of a carbonic acid is effected by the mutual decomposition of a carbonate with a vegetable acid or an acid salt. Of the carbonates used, the preference is usually given to the bicarbonate of potassa or soda, seldom only the officinal carbonate of magnesia or monocarbonate of soda are employed; carbonate of potassa is entirely unfit to enter into such a combination, owing to its deliquescence.

Among the acids, recourse is had to the tartaric and citric, they being the only two officinal acids suitable for such a preparation; sometimes they are replaced by bitartrate of potassa, the object of which is to have the evolution of carbonic acid going on in the stomach.

In the United States and England, the carbonate and the acid are usually kept in two separate papers, distinguished by their blue and white color, while on the continent of Europe, for its greater convenience, a mixture of the two is habitually employed and is even officinal in most of the continental Pharmacopœias.

The U. S. Pharmacopœia has no officinal formula for any of these powders; however it is customary for the apothecary to prepare two different kinds, one under the name of "*soda powders*" which shall contain for one dose 30 grains of bicarbonate of soda in a blue paper, and 25 grains of tartaric acid in a white one. The "*Seidlitz powders*," intended for a slight laxative, require 2 drachms of tartrate of soda and potassa with 10 grains of bicarbonate of soda in a blue, and 30 grains of tartaric acid in a white paper. These powders became officinal in the Prussian Pharmacopœia under the name of English effervescing powders, (Pulvis aerŏphorus Anglicus) and are put up in the greatest part of Europe in accordance with this formula. It has been proposed by Dr. Mohr to substitute tartrate of soda for Rochelle salt, from which tartaric acid precipitates cream of tartar. Sometimes, especially in France, where such formula have originated and were published, the Rochelle salt is partly or wholly replaced by sulphate of soda, and sulphate of magnesia, and the powders thus prepared indiscriminately sold under the name of "poudre de Sedlitz," from which they chiefly differ by their bitter taste, the aperient properties being about alike. If these powders are put up in an air-dry state, they keep admirably well for any length of time. Of the British Pharmacopæias, the Edinburgh and Dublin give directions for similar preparations under the name of pulveres effervescentes citrati, which are made of citric, instead of tartaric acid. Under the name of simple effervescing or Seltzer powder, Dorvault directs 4 grammes (61.7 grs.) of bicarbonate of soda in a blue, and the same quantity of tartaric acid in a white paper, which, when dissolved in a wine bottle full of water containing 1½ or 2 oz. of syrup of currants or lemon, form a very refreshing drink. There are many similar formulæ for "effervescing lemonade," usually consisting of bicarbonate of soda, which for its cheapness is preferred to the potassa salt, mixed, with a certain quantity of sugar, flavored with oil of lemon in one paper, and of tartaric or citric acid done up in another one. The chief object in making such powders is to obtain their refrigerent qualities, and the acid is therefore taken somewhat in excess to assure of the entire decomposition of the carbonate and to procure a quantity of an acid salt, either bitartrate or bicitrate, which by its agreeable acidulous taste adds much to the pleasant and refreshing properties. Sometimes, however, the physician desires an ant-acid effect at the same time with the refrigerant action of the carbonic acid, and increases the quantity of the alkaline carbonate sometimes far beyond the power of saturation of the acid employed

An effervescing powder is also occasionally made, consisting of bicarbonate of ammonia, and of citric acid, and thus forms a pleasant substitute for the solution of citrate of ammonia of the London College, inasmuch as with each dose beside the ammonia, a corresponding quantity of carbonic acid is administered; 20 grains of crystallized citric acid will saturate 18 grains of the bicarbonate of ammonia, the quantity of which, however, may be enlarged if a more decided action of the ammonia is wanted.

Sometimes, though as yet not very often, if Seidlitz powders are excepted, effervescing powders are used to cover the unpleasant taste of some medicines; in this view they are mostly used in connection with Rochelle, Epsom, and Glauber's salt, the bitterness of which salt is to a certain degree masked, particularly if taken in connection with lemon syrup. Meirieu has proposed to administer sulphate of quinine in this manner, by mixing 1 decigramme of it (1.5 grains) with 1 gramme of tartaric acid, and in another paper 1.2 grammes of bicarbonate of soda and 8 grammes sugar; the quantity of the acid just suffices to convert the soda into the neutral tartrate, and to render the quinine easily soluble, which is intended to be taken with its bitterness masked by the sugar and the evolved carbonic acid. A ferruginous effervescing powder has been proposed by Colombat, and the formula for it published by Dorvault. The powders are made by mixing 2 grms. (grs. 30.87) sulphate of iron, 6 grms. (grs. 92.60) tartaric acid and 12 grms. (3 drachms) sugar, and dividing the mixture into 12 powders, which are done up in white paper; each of the blue papers contains 5 grs. of bicarbonate of soda and 15 grs. of sugar. A reaction at first takes place between the iron and soda salt, resulting in some sulphate of soda and bicarbonate of the protoxide of iron, which is decomposed together with the rest of the bicarbonate of soda by the tartaric acid; the acid is just within the fraction of not quite 2 grains for the 12 powders, sufficient to produce tartrate of the protoxide of iron and bitartrate of soda. If the assertion of many practitioners be correct, that iron exhibits the most useful and reliable action in the animal body when administered in the state of protoxide, the above prescription might perhaps be found very beneficial and claim the attention of physicians. Tartrate of protoxide of iron is but little soluble in water, and it might perhaps be thought objectionable on that account; it remains to be seen, however, whether bitartrate of soda, or the carbonic acid does not act as a solvent, or whether the digestive liquids do not easily decompose it, so as to assimilate the iron. If the pure sulphate of the protoxide is mixed with tartaric acid, the mixture keeps admirably well, so far at least as may be judged from the color, and in making such an effervescing draught, the iron will consequently reach the stomach before it can be oyxdized to sequioxide by the influence of the atmosphere.

In the preceding I have taken a cursory view of the various kinds of those effervescing powders, which are put up with the acid and the carbonate in different parcels, and will now proceed to the principal object of this paper, that of drawing attention to that very convenient form of the same kind of powders, which differ from the former in containing all the materials necessary for the evolution of carbonic acid gas mixed into one uniform powder. Of these effervescing powders, Wood and Bache's Dispensatory has on page 54 the following short notice: "Tartaric acid, dried by a gentle heat and then mixed in due proportion with bicarbonate of soda, forms a good effervescing powder, a teaspoonful of which, stirred into a tumbler of water forms the dose. The mixture must be kept in well stopped vials." Every body who has been making such powders, will have experienced that such a mixture, no matter how closely stopped it may be kept, will spoil and even become moist. Professor Otto has made some experiments and discovered some interesting facts with regard to this phenomenon, an account of which he has published in the Annalen der Chemie and Pharmacie, xvii, 378, of which I will give a short abstract. A mixture of equal parts of bicarbonate of soda and tartaric acid becomes moist when introduced into well stopped vials, and this change takes place the quicker, the better the air is excluded; it keeps better if the vials are simply covered with paper; but if the powder is kept in ordinary paper boxes, he has never seen it to spoil. Experiments of Bosse have shown that such a mixture loses weight from the expulsion of carbonic acid, which loss is greater, the better the powder is secured from contact with the air. This decomposition is introduced by a portion of moisture which the mixture contains, and which must evaporate on the air, to keep the powder unaltered. But if both ingredients are dried before mixing at a temperature between 120 and 145° F. the tartaric acid loses nothing, but the bicarbonate of soda $1\frac{1}{2}$ per cent.; if the mixture is now made and introduced into a well stopped vial, it has commenced to decompose after the lapse of 24 hours and the decomposition progresses rapidly. But if the soda salt was dried at 167° F. (60° R.) the mixture was unaltered after having been kept 12 days in a closed vial. It is the water of combination, and not hygroscopic water that introduces the change, though damp favors it; this water is set free at the formation of tartrate of soda, and causes the moisture and liquefaction, it must be allowed to evaporate; that takes place easily from the fine powder at the tension of a low temperature, and for this reason the alteration is so little that it is without any consequence. Such a mixture which has been in contact with the air for some time and is still unaltered, commences to change as soon as it is introduced into a stopped vial. If the powder is directed to be kept in a vial, it might be more advisable not to dry the ingredients, but the finely powdered mixture. In no case, however, not even with the greatest care could the powder be kept for many years.

These remarks apply next to the effervescing powder of the Prussian Pharmacopœia, with which the experiments were made; it is prepared of 4 parts of bicarbonate of soda, 3 parts of tartaric acid, and 7 parts of white sugar. Immediately on throwing this powder into water, effervescence takes place violently. Other preparations, however, generate the carbonic acid slowly, even so slow that the greater part of the reaction takes place in the stomach. By this means quite a quantity of carbonic acid can be introduced into the stomach at a single dose, and is there mostly generated by degrees. Vogler gives the following formula: Exsiccated carbonate of soda ʒij, bicarbonate of potassa ʒij, sugar ℥ss. If three drachms of bicarbonate of soda are substituted in this formula for the exsiccated soda, a larger amount of carbonic acid will be evolved. But as bitartrate of soda is a heavy salt and only slightly soluble in cold water, the idea suggests itself of looking for a comparatively more soluble salt, to take the place of the former in such a combination. Such a salt we find in the bitartrate of soda, which is much easier soluble in water than cream of tartar, but does not act so violently on the carbonate as the free acid, especially not if cold water is used for the menstrum. Bicarbonate and bitartrate of soda must be used in the proportion of 9:20 to furnish a neutral salt. If the ingredients are pulverized separately and afterwards mixed with finely powdered sugar, the mixture will keep very well in a paper box, with no other precaution except to keep it away from dampness.

In a similar manner effervescing powders may be prepared with bicarbonate of ammonia, of which 14 parts are necessary to completely saturate 15 parts of tartaric acid, so as to form the neutral tartrate of ammonia, or 30 parts of the acid are required if the formation of bitartrate of ammonia is desired. 7 Parts of bicarbonate of ammonia will saturate 18½ parts of bitartrate of potassa, and 3 parts of the carbonate, 8¼ parts of bitartrate of soda, in the first case forming the tartrate of ammonia and potassa, in the latter tartrate of ammonia and soda. The same results may be arrived at, if bitartrate of ammonia is used, and it may suffice simply to state here the required proportions:

16 Parts of it saturate 7 parts of bicarbonate of ammonia, forming neutral tartrate of ammonia.

2 Parts of it saturate 1 part of bicarbonate of soda, forming tartrate of soda and ammonia.

4 Parts of it saturate 2½ parts of bicarbonate of potassa, forming tartrate of potassa and ammonia.

3 Parts of it saturate 1 part of dry carbonate of soda, forming tartrate of soda and ammonia.

An effervescing powder containing magnesia was formerly more extensively used in Europe than at present; it consisted of carbonate of magnesia ʒij, tartaric acid ʒss, sugar ʒi, oil of lemon gtt. iij. An effective and very pleasant cathartic may be prepared from the following ingredients: carbonate of magnesia, citric acid, sugar, *aa* ʒij to ʒiij, oil of lemon gtt. ij. The ingredients are to be separately rubbed into a fine powder and then mixed. Thus extemporaneously prepared and soon taken, it makes a pleasant drink and is a good cathartic. The powder is to be stirred into about a half a pint of water, when chemical reaction will instantly commence; when it has fairly set in it should be taken at once. I have omitted to make experiments with regard to its keeping. It remains to be seen, and experiments ought to be made, whether it may be preserved unaltered for some time according to the plan suggested by Professor Otto. As citric acid contains much water of crystalization (4 equiv.) it might probably in this case be advisable to first expel the same by exposure to heat, and after it has become quite cool to mix it with the other ingredients previously finely powdered. It might be of great service upon voyages in cases where saving of space is of some account, and where bottles cannot be well packed ; it would combine smallness of bulk and portability, and for these reasons would be preferable to the officinal solution. Its great advantage over the soluble citrate of magnesia would be its cheapness and its far more refreshing taste, produced by the carbonic acid set free and the citric acid still in solution. The citric acid in the formula given may be augmented, but as the reaction does not take place at once, and as it is drank before the saturation is completed, there is no reason for doing so; the solution will still hold a sufficiency of free citric acid to cover the taste of the magnesia salt, and render the draught quite a refreshing one ; the chemical reaction is completed in the stomach.

If the administration of an iron salt is intended, Colombat's formnla given above may be used, and all the ingredients mixed into one powder ; but it must be carried in mind that this results in a *tartrate* of the protoxide of iron. As it has always been a desideratum how to administer a carbonate of the protoxide of iron in an unaltered state, and as even Vallet's mass becomes oxidized, the following mode of administration is suggested. Sulphate of iron ʒiss, tartaric acid ℈ijss, dry carbonate of soda ℈iijss, sugar ʒiij. The chalybeate waters contain usually the carbonate of protoxide of iron, dissolved by an excess of carbonic acid, besides some alkaline salts. Physicians value these waters, because they are usually better adapted to the digestive organs and are easier assimilated, consequently of a quicker and more reliable action on the human frame than the ordinary ferruginous preparations. These properties are doubtless due to the presence of that mild acid, the carbonic and probably to the aperient salts, both of which combine their action with that of the iron. In the formula given above sufficient carbonic acid is generated to convert the iron salt into a carbonate of the protoxide, and keep the same in solution. Thrown into a bottle filled with water, a clear solution is obtained, from which on exposure to the air sesquioxide of iron is separated. If a brisker effervescence is desired, the acid and soda may be increased in due proportion, or instead of the latter, 5½ scruples of bicarbonate of soda substituted, when the mixture will contain double the amount of carbonic acid. A little sulphate of soda, corresponding with the quantity of iron will be formed, and the neutral tartrate of soda which does not decompose the dissolved carbonate of protoxide of iron. The quantity of these salts formed is but small, only large enough as is necessary for obtaining a sufficiency of carbonic acid ; thus an opportunity is offered to combine with the powder such substances as may be thought necessary to increase its effect or direct it to a certain point ; such substances of course must not interfere with the carbonate of iron. The teaspoonful of the above chalybeate effervescing powder contains about 18 grs. of sulphate, equal to 7½ grs. of carbonate of protoxide of iron. With regard to the sulphate of iron I would here yet remark, that even not the finest crystals ought to be used for such a preparation if stability is desired. Without entering into details at present it may suffice to say, that this salt keeps best, by itself and mixed with other powders, if it has been precipitated from a concentrated solution by strong alcohol, well washed with the same, and afterward well dried in the open air, spread out in thin layers on bibulons paper.

My investigation with reference to the administration of quinine has not been completed yet, but the few experiments made convince me that it is a very good mode to administer quinine and cinchonine in an effervescing powder, made with citric acid and sweetened with sugar, previously rubbed up with the yellow skin of fresh orange peel. This corrects the taste better than lemon, cloves, or any other aromatic that I have tried. The bitterness seems to be less perceptible, when the effervescing powder affords a nearly neutral mixture, and a sufficiency of the orange skin has been added to impart a high and agreeable flavor to the draught.

*Administration of Ammonia.*—Bicarbonate of ammonia is devoid of the ammoniacal smell, and, although, having still a somewhat pungent taste, may be given in almost any form, even the powder and solution hardly requiring more than a little sweetening to render it pleasant to take. If the full effects of ammonia are quickly desired, we have no other preparation for such a purpose, but the various preparations of caustic ammonia and the officinal sesquicarbonate. This latter one only can be made into pills, but is usually given in solution, like spiritus and aqua ammoniæ; their taste and smell, however, are so very pungent and penetrating, that they cannot be covered or masked, either by aromatics or mucilages. As they are valuable remedies in scarlet fever, the small children, for whom they are prescribed, often object to taking them. We have, I believe, a much more convenient and pleasant form to secure the effects of ammonia without incurring its penetrativeness; we may arrive at this end in a somewhat similar way as above, in trying to obtain the properties of carbonate of protoxide of iron. If a solution of the neutral tartrate of ammonia is mixed with a solution of carbonate of potassa, an ammoniacal smell will soon be perceived, and a glass rod, moistened with muriatic acid, will evolve thick white vapors if held above the surface of the liquid. An interchange has taken place, a double decomposition, by which tartrate of potassa and carbonate of ammonia, has been formed.

If bitartrate of ammonia is mixed with a solution of carbonate of potassa, an effervescence will take place, and the formation of a double tartrate of the two bases; a further addition of carbonate of potassa will displace the ammonia from its combination, which, with the carbonic acid of the potassa salt and that still held back by the solution, combines to bicarbonate or sesquicarbonate of ammonia. But, if, after the saturation of the bitartrate of ammonia with carbonate of potassa, the carbonic acid is expelled by a moderate heat, or, if the double tartrate of potassa and ammonia be taken at once, the subsequent addition of carbonate of potassa causes the formation of monocarbonate of ammonia. Bicarbonate of potassa used in place of the carbonate, will, in all cases, cause the same result, that is, the production of bicarbonate of ammonia. Consequently, if we want the formation of monocarbonate of ammonia, we have to bring in contact neutral tartrate of ammonia, or its double salt with tartrate of potassa, and carbonate of potassa; as this latter, however, cannot be dispensed in form of powder, and as it dissolves in water too easily, and for this reason acts too quickly on the tartrate so as to form carbonate of ammonia, it is not fit to answer the purpose of having the reaction going on in the stomach so that the powder may pass the organs of smell and taste without exhibiting anything but a salinous taste; the dry carbonate of soda then takes its place, on account of its similar action and its slower solubility; even carbonate of lime might be used in some cases; the powder should be mixed in syrup and given at once, but care should be taken not to direct an excess of carbonate of soda, which might develope its caustic properties in the stomach. Tartrate of ammonia, with which I have experimented a short time ago, I believe to be a far more stable salt than is usually supposed, but other salts such as the sulphate and muriate can be used in lieu thereof; it should, however, never be left out of sight that with these powders a double decomposition takes place, and that, for instance, a mixture of carbonate of lime and sal ammonia would be inadmissible, on account of the formation of chloride of calcium. One part of exsiccated carbonate of soda would suffice for one and three-quarter parts of the neutral tartrate, one and a quarter of sulphate and one part of muriate of ammonia.

Whenever medicine is administered, it ought not only to be prepared nicely, but appearance and taste ought to be made as agreeable as possible, so as to please the eye, and, if possible, the palate of the invalid. Very often has the pharmaceutist occasion to take notice of some behavior of medicines in this regard, often he may be interested sufficiently to make some experiments for the sake of information, and thus arrive at some satisfactory results. We know sugar has been tried to mask the taste of disagreeable medicines, aromatics and spirits have been tried, but for some in vain. The effervescing powder seemed to be one means that had not been sufficiently tried yet, and a desire to find a more pleasant way for administering ammonia, has also lead to the experiments with iron to discover a way to administer it in the state in which it occurs in most of the chalybeate springs. These are the reasons for having undertaken these investigations; if the result and the suggestions expressed will inspire others to a trial of pushing forward in this direction, the results might probably be very satisfactory.

